# *N*,*N*′-Bis(phenyl­carbamo­thio­yl)benzene-1,3-dicarboxamide

**DOI:** 10.1107/S1600536813020163

**Published:** 2013-08-03

**Authors:** Zainab Ngaini, Maya Asyikin Mohd Ariff, Wan Sharifatun Handayani Wan Zullkiplee, Hasnain Hussain, Mohd Mustaqim Rosli

**Affiliations:** a Department of Chemistry, Faculty of Resource Science and Technology, Universiti Malaysia Sarawak, 94300 Kota Samarahan, Sarawak, Malaysia; b Department of Molecular Biology, Faculty of Resource Science and Technology, Universiti Malaysia Sarawak, 94300 Kota Samarahan, Sarawak, Malaysia; c X-ray Crystallography Unit, School of Physics, Universiti Sains Malaysia, 11800 USM, Penang, Malaysia

## Abstract

The asymmetric unit of the title compound, C_22_H_18_N_4_O_2_S_2_, contains two mol­ecules. In one of them, the dihedral angles between the central benzene ring and the phenyl rings are 16.97 (8) and 20.97 (8)°, while the phenyl rings make a dihedral angle of 37.87 (8)°. In the other mol­ecule, the corresponding values are 34.92 (7), 53.90 (7) and 60.68 (8)°, respectively. In each mol­ecule, two intra­molecular N—H⋯O hydrogen bonds generate *S*(6) rings and a short C—H⋯S contact also occurs. In the crystal, N—H⋯S, N—H⋯O, C—H⋯O and C—H⋯S inter­actions link the mol­ecules into a three-dimensional network.

## Related literature

For biological applications of benzimidazole derivatives, see: Madan *et al.* (1991 ▶); Fernandez *et al.* (2005 ▶); Kucukguzel *et al.* (2008 ▶); Saeed *et al.* (2009 ▶). For biological properties of thioureas, see: Rauf *et al.* (2009 ▶).
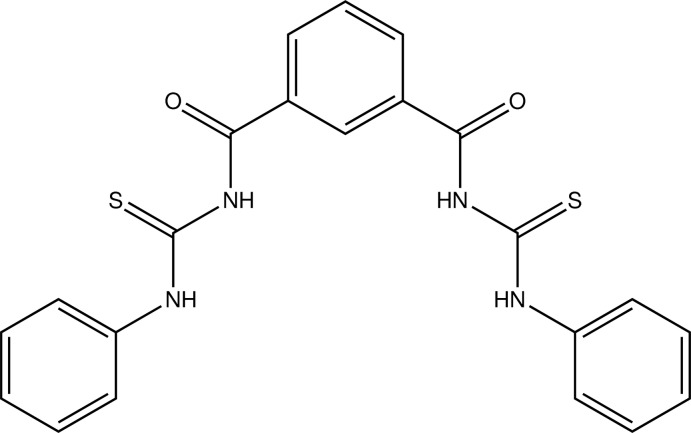


## Experimental

### 

#### Crystal data

C_22_H_18_N_4_O_2_S_2_
*M* *_r_* = 434.52
Triclinic, 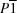

*a* = 11.1812 (2) Å
*b* = 11.5623 (2) Å
*c* = 16.4471 (2) Å
α = 101.420 (1)°
β = 98.127 (1)°
γ = 101.316 (1)°
*V* = 2007.43 (6) Å^3^
*Z* = 4
Mo *K*α radiation
μ = 0.29 mm^−1^
*T* = 100 K
0.47 × 0.33 × 0.11 mm

#### Data collection

Bruker APEX DUO CCD diffractometer
Absorption correction: multi-scan (*SADABS*; Bruker, 2009 ▶) *T*_min_ = 0.876, *T*_max_ = 0.968
41276 measured reflections
14580 independent reflections
11642 reflections with *I* > 2σ(*I*)
*R*_int_ = 0.034

#### Refinement

*R*[*F* ^2^ > 2σ(*F* ^2^)] = 0.044
*wR*(*F* ^2^) = 0.122
*S* = 1.03
14580 reflections
573 parameters
H atoms treated by a mixture of independent and constrained refinement
Δρ_max_ = 0.53 e Å^−3^
Δρ_min_ = −0.28 e Å^−3^

### 

Data collection: *APEX2* (Bruker, 2009 ▶); cell refinement: *SAINT* (Bruker, 2009 ▶); data reduction: *SAINT*; program(s) used to solve structure: *SHELXTL* (Sheldrick, 2008 ▶); program(s) used to refine structure: *SHELXTL*; molecular graphics: *SHELXTL*; software used to prepare material for publication: *SHELXTL* and *PLATON* (Spek, 2009 ▶).

## Supplementary Material

Crystal structure: contains datablock(s) I, New_Global_Publ_Block. DOI: 10.1107/S1600536813020163/hb7103sup1.cif

Structure factors: contains datablock(s) I. DOI: 10.1107/S1600536813020163/hb7103Isup2.hkl

Supplementary material file. DOI: 10.1107/S1600536813020163/hb7103Isup3.cml

Additional supplementary materials:  crystallographic information; 3D view; checkCIF report

## Figures and Tables

**Table 1:** Hydrogen-bond geometry (Å, °)

*D*—H⋯*A*	*D*—H	H⋯*A*	*D*⋯*A*	*D*—H⋯*A*
N1*A*—H1*NA*⋯O1*A*	0.83 (2)	1.976 (19)	2.6722 (16)	140.9 (19)
N2*A*—H2*NA*⋯S2*B* ^i^	0.85 (2)	2.59 (2)	3.4201 (12)	165.0 (19)
N3*A*—H3*NA*⋯O2*B* ^ii^	0.864 (19)	2.31 (2)	2.9715 (15)	133.3 (18)
N4*A*—H4*NA*⋯O2*A*	0.90 (2)	1.86 (2)	2.6064 (17)	138.2 (18)
N1*B*—H1*NB*⋯O1*B*	0.86 (2)	1.88 (2)	2.6248 (17)	144.5 (19)
N2*B*—H2*NB*⋯S1*B* ^iii^	0.834 (19)	2.71 (2)	3.4961 (12)	158.3 (19)
N3*B*—H3*NB*⋯S1*A* ^i^	0.84 (2)	2.62 (2)	3.4336 (12)	163.0 (18)
N4*B*—H4*NB*⋯O2*B*	0.87 (2)	1.92 (2)	2.6543 (16)	141.1 (19)
C5*A*—H5*AA*⋯S1*A*	0.95	2.51	3.1910 (16)	129
C1*B*—H1*BA*⋯S1*B*	0.95	2.68	3.2693 (15)	121
C4*B*—H4*BA*⋯O2*A* ^iv^	0.95	2.55	3.4819 (18)	165
C10*B*—H10*B*⋯S2*A* ^v^	0.95	2.84	3.4570 (15)	123
C11*B*—H11*B*⋯S2*A* ^v^	0.95	2.85	3.4687 (15)	123
C14*A*—H14*A*⋯O2*B* ^ii^	0.95	2.35	3.2700 (17)	164
C14*B*—H14*B*⋯O1*A* ^ii^	0.95	2.36	3.2897 (17)	165

Symmetry codes: (i) 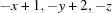; (ii) 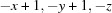; (iii) 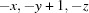; (iv) 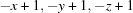; (v) 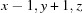.

**Fig. 1.**
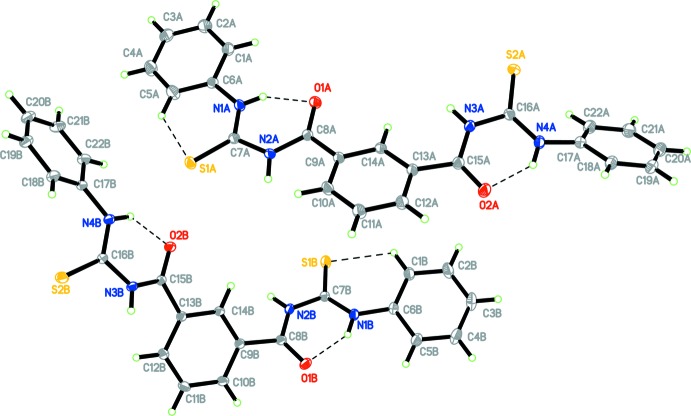
The asymmetric unit of the title compound, showing 50% probability displacement ellipsoids.

**Fig. 2.**
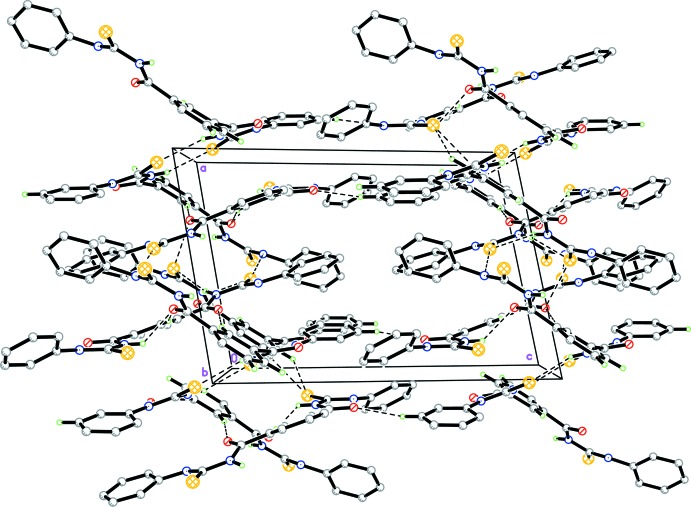
The crystal packing of (I). Dashed lines indicate hydrogen bonds. H atoms not involved in the hydrogen bond interactions have been omitted for clarity.

## supplementary crystallographic information

### 1. Comment

Thiourea derivatives play an important roles in many biological activities (Madan *et al.*, 1991) such as antimycobacterial agent (Fernandez *et al.*, 2005), antiviral (Kucukguzel *et al.*, 2008), and antibacterial (Saeed *et al.*, 2009). As part of our studies in this area, we now describe the title compound, which contains two thiourea groups from the reaction of 3-acetylbenzoyl isothiocyanate with aniline.

There are two molecules in the asymmetric unit (Fig. 1) of the title compound. For molecule A, the dihedral angles between the aldehyde benzene (C9A—C14A) and the phenyl rings (C1A—C6A & C17A—C22A) are 16.97 (8) and 20.97 (8)°, respectively while the two phenyl rings make a dihedral angle of 37.87 (8)°. In the molecule B, corresponding values are 34.92 (7), 53.90 (7)° and 60.68 (8)° respectively.

In each molecule, three set of S(6) hydrogen rings motif can be observe involving the N—H···O and C—H···S intramolecular interactions. An extensive intermolceular interactions of N—H···S, N—H···O, C—H···O and C—H···S link the molecules into a three-dimensional network (Fig. 2, Table 1).

### 2. Experimental

Isophthaloyl dichloride (1 mmol) in 15 ml of dry acetone was added drop wise to a suspension of potassium thiocyanate (2 mmol) in 15 ml of dry acetone. The mixture was stirred for 1 h at room temperature and the white potassium chloride (KCl) was filtered. Aniline (2 mmol) in dry acetone (15 ml) was added into the filtrate and heated under reflux for 7 h. The mixture was cooled to room temperature and filtered. The filtrate was poured into ice in a beaker to form solid. The crude was filtered, washed with ethanol and recrystallized from ethanol-acetonitrile (1:1) solution to yield colourless blocks.

### 3. Refinement

N bound H atoms were located from difference Fourier maps and freely refined. The remaining H atoms were positioned geometrically and refined using a riding model with with C–H = 0.95 Å and *U*_iso_(H) = 1.2*U*_eq_(C).

### Crystal data

**Table:**

C_22_H_18_N_4_O_2_S_2_	*Z* = 4
*M**_r_* = 434.52	*F*(000) = 904
Triclinic, *P*1	*D*_x_ = 1.438 Mg m^−^^3^
Hall symbol: -P 1	Mo *K*α radiation, λ = 0.71073 Å
*a* = 11.1812 (2) Å	Cell parameters from 9969 reflections
*b* = 11.5623 (2) Å	θ = 2.6–32.6°
*c* = 16.4471 (2) Å	µ = 0.29 mm^−^^1^
α = 101.420 (1)°	*T* = 100 K
β = 98.127 (1)°	Block, colourless
γ = 101.316 (1)°	0.47 × 0.33 × 0.11 mm
*V* = 2007.43 (6) Å^3^	

### Data collection

**Table:**

Bruker APEX DUO CCD diffractometer	14580 independent reflections
Radiation source: fine-focus sealed tube	11642 reflections with *I* > 2σ(*I*)
Graphite monochromator	*R*_int_ = 0.034
φ and ω scans	θ_max_ = 32.7°, θ_min_ = 1.9°
Absorption correction: multi-scan (*SADABS*; Bruker, 2009)	*h* = −16→16
*T*_min_ = 0.876, *T*_max_ = 0.968	*k* = −17→17
41276 measured reflections	*l* = −24→24

### Refinement

**Table:**

Refinement on *F*^2^	Primary atom site location: structure-invariant direct methods
Least-squares matrix: full	Secondary atom site location: difference Fourier map
*R*[*F*^2^ > 2σ(*F*^2^)] = 0.044	Hydrogen site location: inferred from neighbouring sites
*wR*(*F*^2^) = 0.122	H atoms treated by a mixture of independent and constrained refinement
*S* = 1.03	*w* = 1/[σ^2^(*F*_o_^2^) + (0.060*P*)^2^ + 0.666*P*] where *P* = (*F*_o_^2^ + 2*F*_c_^2^)/3
14580 reflections	(Δ/σ)_max_ = 0.002
573 parameters	Δρ_max_ = 0.53 e Å^−^^3^
0 restraints	Δρ_min_ = −0.28 e Å^−^^3^

### Special details

**Table:**

Geometry. All e.s.d.'s (except the e.s.d. in the dihedral angle between two l.s. planes) are estimated using the full covariance matrix. The cell e.s.d.'s are taken into account individually in the estimation of e.s.d.'s in distances, angles and torsion angles; correlations between e.s.d.'s in cell parameters are only used when they are defined by crystal symmetry. An approximate (isotropic) treatment of cell e.s.d.'s is used for estimating e.s.d.'s involving l.s. planes.
Refinement. Refinement of *F*^2^ against ALL reflections. The weighted *R*-factor *wR* and goodness of fit *S* are based on *F*^2^, conventional *R*-factors *R* are based on *F*, with *F* set to zero for negative *F*^2^. The threshold expression of *F*^2^ > σ(*F*^2^) is used only for calculating *R*-factors(gt) *etc*. and is not relevant to the choice of reflections for refinement. *R*-factors based on *F*^2^ are statistically about twice as large as those based on *F*, and *R*- factors based on ALL data will be even larger.

### Fractional atomic coordinates and isotropic or equivalent isotropic displacement parameters (Å^2^)

**Table:**

	*x*	*y*	*z*	*U*_iso_*/*U*_eq_	
S1A	0.56880 (4)	0.73303 (3)	−0.12090 (2)	0.01838 (7)	
S2A	0.87429 (3)	0.08533 (3)	0.22615 (2)	0.01788 (7)	
O1A	0.70303 (10)	0.43088 (9)	−0.02345 (6)	0.01691 (19)	
O2A	0.84638 (12)	0.45365 (10)	0.37157 (6)	0.0243 (2)	
N1A	0.58550 (12)	0.49816 (11)	−0.15304 (7)	0.0158 (2)	
N2A	0.63967 (11)	0.60868 (10)	−0.01410 (7)	0.0149 (2)	
N3A	0.85008 (11)	0.31200 (10)	0.25405 (7)	0.0151 (2)	
N4A	0.86694 (12)	0.23330 (11)	0.37280 (7)	0.0174 (2)	
C1A	0.55898 (15)	0.34542 (13)	−0.27844 (9)	0.0203 (3)	
H1AA	0.5931	0.2985	−0.2440	0.024*	
C2A	0.52227 (17)	0.29874 (14)	−0.36505 (9)	0.0255 (3)	
H2AA	0.5314	0.2201	−0.3895	0.031*	
C3A	0.47246 (16)	0.36650 (15)	−0.41579 (9)	0.0237 (3)	
H3AA	0.4475	0.3348	−0.4750	0.028*	
C4A	0.45953 (17)	0.48052 (15)	−0.37945 (9)	0.0273 (3)	
H4AA	0.4254	0.5271	−0.4142	0.033*	
C5A	0.49557 (16)	0.52866 (15)	−0.29272 (9)	0.0257 (3)	
H5AA	0.4858	0.6072	−0.2685	0.031*	
C6A	0.54596 (13)	0.46072 (12)	−0.24196 (8)	0.0148 (2)	
C7A	0.59717 (12)	0.60539 (12)	−0.09913 (8)	0.0139 (2)	
C8A	0.69641 (12)	0.52843 (12)	0.01927 (8)	0.0137 (2)	
C9A	0.74873 (12)	0.56819 (12)	0.11167 (8)	0.0140 (2)	
C10A	0.77808 (14)	0.68980 (12)	0.15500 (8)	0.0178 (2)	
H10A	0.7681	0.7507	0.1251	0.021*	
C11A	0.82180 (15)	0.72217 (13)	0.24174 (9)	0.0212 (3)	
H11A	0.8402	0.8048	0.2713	0.025*	
C12A	0.83832 (14)	0.63308 (13)	0.28471 (8)	0.0197 (3)	
H12A	0.8658	0.6548	0.3442	0.024*	
C13A	0.81496 (13)	0.51154 (12)	0.24138 (8)	0.0148 (2)	
C14A	0.76909 (12)	0.47850 (12)	0.15455 (8)	0.0139 (2)	
H14A	0.7519	0.3960	0.1248	0.017*	
C15A	0.83784 (13)	0.42367 (12)	0.29426 (8)	0.0162 (2)	
C16A	0.86424 (12)	0.21278 (12)	0.28948 (8)	0.0143 (2)	
C17A	0.86565 (13)	0.15044 (13)	0.42592 (8)	0.0167 (2)	
C18A	0.92475 (14)	0.19911 (14)	0.50987 (8)	0.0196 (3)	
H18A	0.9674	0.2820	0.5275	0.024*	
C19A	0.92110 (15)	0.12619 (14)	0.56758 (9)	0.0218 (3)	
H19A	0.9603	0.1597	0.6249	0.026*	
C20A	0.86063 (15)	0.00499 (14)	0.54201 (9)	0.0225 (3)	
H20A	0.8603	−0.0454	0.5812	0.027*	
C21A	0.80024 (15)	−0.04280 (14)	0.45842 (10)	0.0228 (3)	
H21A	0.7577	−0.1258	0.4411	0.027*	
C22A	0.80140 (14)	0.02947 (13)	0.40006 (9)	0.0198 (3)	
H22A	0.7589	−0.0033	0.3434	0.024*	
S1B	0.04057 (4)	0.37065 (3)	0.07939 (2)	0.01870 (8)	
S2B	0.48059 (4)	1.19306 (3)	−0.11559 (2)	0.01956 (8)	
O1B	0.13462 (11)	0.75257 (9)	0.24757 (6)	0.0201 (2)	
O2B	0.30600 (9)	0.81658 (9)	−0.08677 (6)	0.01593 (18)	
N1B	0.13188 (11)	0.52187 (11)	0.23333 (7)	0.0154 (2)	
N2B	0.08889 (11)	0.60964 (10)	0.12230 (7)	0.0145 (2)	
N3B	0.36721 (11)	1.02400 (10)	−0.04957 (7)	0.0131 (2)	
N4B	0.45042 (11)	0.95054 (10)	−0.16480 (7)	0.0139 (2)	
C1B	0.18586 (14)	0.33048 (13)	0.25490 (9)	0.0195 (3)	
H1BA	0.1860	0.3028	0.1966	0.023*	
C2B	0.21509 (16)	0.26110 (14)	0.31178 (10)	0.0255 (3)	
H2BA	0.2354	0.1857	0.2920	0.031*	
C3B	0.21489 (16)	0.30126 (15)	0.39736 (10)	0.0269 (3)	
H3BA	0.2337	0.2528	0.4356	0.032*	
C4B	0.18724 (16)	0.41160 (16)	0.42659 (9)	0.0266 (3)	
H4BA	0.1882	0.4396	0.4851	0.032*	
C5B	0.15812 (15)	0.48148 (14)	0.37044 (9)	0.0220 (3)	
H5BA	0.1392	0.5574	0.3905	0.026*	
C6B	0.15657 (13)	0.44021 (12)	0.28430 (8)	0.0155 (2)	
C7B	0.09048 (12)	0.50324 (12)	0.15029 (8)	0.0139 (2)	
C8B	0.11636 (12)	0.72663 (12)	0.16982 (8)	0.0140 (2)	
C9B	0.12775 (12)	0.82365 (11)	0.12203 (8)	0.0133 (2)	
C10B	0.08163 (13)	0.92559 (12)	0.14973 (8)	0.0168 (2)	
H10B	0.0379	0.9289	0.1953	0.020*	
C11B	0.09979 (13)	1.02205 (12)	0.11061 (9)	0.0173 (2)	
H11B	0.0644	1.0893	0.1272	0.021*	
C12B	0.16985 (13)	1.01978 (12)	0.04711 (8)	0.0151 (2)	
H12B	0.1838	1.0863	0.0210	0.018*	
C13B	0.21967 (12)	0.92008 (11)	0.02166 (8)	0.0126 (2)	
C14B	0.19528 (12)	0.81962 (11)	0.05684 (8)	0.0126 (2)	
H14B	0.2243	0.7494	0.0367	0.015*	
C15B	0.29979 (12)	0.91422 (11)	−0.04329 (7)	0.0127 (2)	
C16B	0.43319 (12)	1.04832 (11)	−0.11312 (7)	0.0126 (2)	
C17B	0.49238 (12)	0.94255 (12)	−0.24293 (8)	0.0135 (2)	
C18B	0.58498 (13)	1.02975 (12)	−0.25842 (8)	0.0168 (2)	
H18B	0.6257	1.0996	−0.2152	0.020*	
C19B	0.61801 (14)	1.01436 (14)	−0.33785 (9)	0.0204 (3)	
H19B	0.6821	1.0737	−0.3483	0.025*	
C20B	0.55823 (14)	0.91338 (14)	−0.40168 (9)	0.0213 (3)	
H20B	0.5787	0.9051	−0.4563	0.026*	
C21B	0.46833 (14)	0.82451 (14)	−0.38515 (9)	0.0211 (3)	
H21B	0.4283	0.7543	−0.4283	0.025*	
C22B	0.43662 (13)	0.83782 (13)	−0.30554 (8)	0.0171 (2)	
H22B	0.3770	0.7755	−0.2939	0.020*	
H1NA	0.6098 (18)	0.4465 (18)	−0.1306 (12)	0.022 (5)*	
H2NA	0.6244 (18)	0.6662 (18)	0.0208 (12)	0.026 (5)*	
H3NA	0.8458 (18)	0.2974 (17)	0.2000 (12)	0.023 (5)*	
H4NA	0.869 (2)	0.310 (2)	0.3996 (13)	0.035 (6)*	
H1NB	0.1426 (19)	0.5963 (19)	0.2599 (13)	0.030 (5)*	
H2NB	0.0724 (19)	0.6022 (18)	0.0702 (12)	0.028 (5)*	
H3NB	0.3662 (18)	1.0839 (19)	−0.0115 (12)	0.027 (5)*	
H4NB	0.4132 (19)	0.8814 (19)	−0.1562 (12)	0.027 (5)*	

### Atomic displacement parameters (Å^2^)

**Table:**

	*U*^11^	*U*^22^	*U*^33^	*U*^12^	*U*^13^	*U*^23^
S1A	0.02852 (18)	0.01355 (14)	0.01349 (14)	0.00880 (13)	0.00118 (12)	0.00214 (11)
S2A	0.02273 (17)	0.01587 (15)	0.01761 (15)	0.00915 (13)	0.00616 (12)	0.00355 (12)
O1A	0.0239 (5)	0.0119 (4)	0.0144 (4)	0.0059 (4)	0.0023 (4)	0.0013 (3)
O2A	0.0430 (7)	0.0183 (5)	0.0123 (4)	0.0121 (5)	0.0031 (4)	0.0018 (4)
N1A	0.0224 (6)	0.0125 (5)	0.0121 (5)	0.0067 (4)	0.0007 (4)	0.0013 (4)
N2A	0.0215 (6)	0.0129 (5)	0.0111 (4)	0.0078 (4)	0.0027 (4)	0.0009 (4)
N3A	0.0207 (6)	0.0138 (5)	0.0113 (4)	0.0054 (4)	0.0027 (4)	0.0027 (4)
N4A	0.0251 (6)	0.0147 (5)	0.0128 (5)	0.0068 (5)	0.0013 (4)	0.0035 (4)
C1A	0.0290 (8)	0.0156 (6)	0.0156 (6)	0.0056 (5)	0.0030 (5)	0.0024 (5)
C2A	0.0392 (9)	0.0191 (7)	0.0163 (6)	0.0097 (6)	0.0028 (6)	−0.0017 (5)
C3A	0.0311 (8)	0.0244 (7)	0.0130 (5)	0.0068 (6)	0.0019 (5)	−0.0002 (5)
C4A	0.0396 (9)	0.0255 (8)	0.0157 (6)	0.0124 (7)	−0.0034 (6)	0.0031 (5)
C5A	0.0376 (9)	0.0212 (7)	0.0157 (6)	0.0137 (6)	−0.0047 (6)	−0.0015 (5)
C6A	0.0167 (6)	0.0145 (6)	0.0115 (5)	0.0027 (5)	0.0016 (4)	0.0008 (4)
C7A	0.0159 (6)	0.0130 (5)	0.0121 (5)	0.0036 (5)	0.0018 (4)	0.0016 (4)
C8A	0.0157 (6)	0.0125 (5)	0.0131 (5)	0.0035 (4)	0.0031 (4)	0.0032 (4)
C9A	0.0168 (6)	0.0123 (5)	0.0129 (5)	0.0045 (5)	0.0022 (4)	0.0020 (4)
C10A	0.0234 (7)	0.0123 (5)	0.0167 (6)	0.0054 (5)	−0.0001 (5)	0.0026 (5)
C11A	0.0303 (8)	0.0128 (6)	0.0170 (6)	0.0054 (5)	−0.0020 (5)	−0.0005 (5)
C12A	0.0274 (7)	0.0150 (6)	0.0139 (5)	0.0053 (5)	−0.0011 (5)	0.0005 (5)
C13A	0.0178 (6)	0.0132 (5)	0.0133 (5)	0.0043 (5)	0.0021 (4)	0.0027 (4)
C14A	0.0168 (6)	0.0114 (5)	0.0129 (5)	0.0036 (4)	0.0023 (4)	0.0014 (4)
C15A	0.0201 (6)	0.0145 (6)	0.0133 (5)	0.0043 (5)	0.0015 (5)	0.0026 (4)
C16A	0.0136 (6)	0.0150 (6)	0.0148 (5)	0.0038 (5)	0.0023 (4)	0.0045 (4)
C17A	0.0195 (6)	0.0177 (6)	0.0148 (5)	0.0073 (5)	0.0040 (5)	0.0047 (5)
C18A	0.0242 (7)	0.0197 (6)	0.0155 (6)	0.0058 (5)	0.0035 (5)	0.0048 (5)
C19A	0.0274 (7)	0.0255 (7)	0.0147 (6)	0.0075 (6)	0.0060 (5)	0.0068 (5)
C20A	0.0264 (7)	0.0256 (7)	0.0215 (6)	0.0083 (6)	0.0114 (5)	0.0126 (6)
C21A	0.0267 (8)	0.0204 (7)	0.0245 (7)	0.0057 (6)	0.0096 (6)	0.0090 (5)
C22A	0.0227 (7)	0.0190 (6)	0.0173 (6)	0.0043 (5)	0.0035 (5)	0.0041 (5)
S1B	0.02706 (18)	0.01150 (14)	0.01460 (14)	0.00189 (13)	−0.00134 (12)	0.00244 (11)
S2B	0.02636 (18)	0.01142 (14)	0.02378 (16)	0.00391 (13)	0.01465 (14)	0.00421 (12)
O1B	0.0331 (6)	0.0156 (5)	0.0122 (4)	0.0055 (4)	0.0074 (4)	0.0026 (3)
O2B	0.0225 (5)	0.0117 (4)	0.0144 (4)	0.0048 (4)	0.0068 (4)	0.0020 (3)
N1B	0.0206 (6)	0.0126 (5)	0.0127 (5)	0.0034 (4)	0.0017 (4)	0.0034 (4)
N2B	0.0202 (6)	0.0131 (5)	0.0106 (4)	0.0036 (4)	0.0032 (4)	0.0036 (4)
N3B	0.0173 (5)	0.0104 (5)	0.0115 (4)	0.0022 (4)	0.0061 (4)	0.0009 (4)
N4B	0.0174 (5)	0.0123 (5)	0.0129 (4)	0.0040 (4)	0.0060 (4)	0.0023 (4)
C1B	0.0236 (7)	0.0176 (6)	0.0178 (6)	0.0058 (5)	0.0015 (5)	0.0055 (5)
C2B	0.0328 (8)	0.0182 (7)	0.0249 (7)	0.0066 (6)	−0.0020 (6)	0.0082 (6)
C3B	0.0314 (8)	0.0237 (7)	0.0231 (7)	0.0004 (6)	−0.0043 (6)	0.0127 (6)
C4B	0.0336 (9)	0.0292 (8)	0.0152 (6)	0.0026 (7)	0.0008 (6)	0.0085 (6)
C5B	0.0296 (8)	0.0216 (7)	0.0147 (6)	0.0066 (6)	0.0024 (5)	0.0043 (5)
C6B	0.0162 (6)	0.0163 (6)	0.0139 (5)	0.0027 (5)	0.0008 (4)	0.0059 (4)
C7B	0.0158 (6)	0.0131 (5)	0.0138 (5)	0.0034 (5)	0.0035 (4)	0.0043 (4)
C8B	0.0160 (6)	0.0128 (5)	0.0140 (5)	0.0033 (5)	0.0052 (4)	0.0031 (4)
C9B	0.0158 (6)	0.0111 (5)	0.0131 (5)	0.0026 (4)	0.0044 (4)	0.0027 (4)
C10B	0.0203 (6)	0.0150 (6)	0.0171 (6)	0.0055 (5)	0.0089 (5)	0.0035 (5)
C11B	0.0200 (6)	0.0127 (5)	0.0218 (6)	0.0065 (5)	0.0092 (5)	0.0039 (5)
C12B	0.0179 (6)	0.0123 (5)	0.0164 (5)	0.0039 (5)	0.0051 (5)	0.0042 (4)
C13B	0.0154 (6)	0.0104 (5)	0.0114 (5)	0.0021 (4)	0.0033 (4)	0.0016 (4)
C14B	0.0155 (6)	0.0104 (5)	0.0119 (5)	0.0035 (4)	0.0033 (4)	0.0014 (4)
C15B	0.0161 (6)	0.0121 (5)	0.0102 (5)	0.0036 (4)	0.0027 (4)	0.0027 (4)
C16B	0.0137 (5)	0.0132 (5)	0.0114 (5)	0.0038 (4)	0.0032 (4)	0.0030 (4)
C17B	0.0153 (6)	0.0141 (5)	0.0123 (5)	0.0057 (5)	0.0039 (4)	0.0027 (4)
C18B	0.0178 (6)	0.0150 (6)	0.0170 (6)	0.0020 (5)	0.0064 (5)	0.0015 (5)
C19B	0.0249 (7)	0.0200 (6)	0.0195 (6)	0.0061 (5)	0.0117 (5)	0.0055 (5)
C20B	0.0252 (7)	0.0244 (7)	0.0159 (6)	0.0069 (6)	0.0096 (5)	0.0036 (5)
C21B	0.0229 (7)	0.0222 (7)	0.0155 (6)	0.0047 (6)	0.0055 (5)	−0.0026 (5)
C22B	0.0173 (6)	0.0150 (6)	0.0179 (6)	0.0029 (5)	0.0062 (5)	0.0003 (5)

### Geometric parameters (Å, º)

**Table:**

S1A—C7A	1.6636 (14)	S1B—C7B	1.6715 (13)
S2A—C16A	1.6611 (14)	S2B—C16B	1.6638 (13)
O1A—C8A	1.2267 (15)	O1B—C8B	1.2317 (15)
O2A—C15A	1.2346 (15)	O2B—C15B	1.2312 (15)
N1A—C7A	1.3449 (16)	N1B—C7B	1.3385 (16)
N1A—C6A	1.4188 (16)	N1B—C6B	1.4215 (17)
N1A—H1NA	0.83 (2)	N1B—H1NB	0.86 (2)
N2A—C8A	1.3780 (17)	N2B—C8B	1.3731 (17)
N2A—C7A	1.4026 (16)	N2B—C7B	1.3986 (17)
N2A—H2NA	0.85 (2)	N2B—H2NB	0.83 (2)
N3A—C15A	1.3711 (17)	N3B—C15B	1.3741 (16)
N3A—C16A	1.4110 (17)	N3B—C16B	1.4032 (15)
N3A—H3NA	0.864 (19)	N3B—H3NB	0.84 (2)
N4A—C16A	1.3385 (16)	N4B—C16B	1.3375 (16)
N4A—C17A	1.4182 (17)	N4B—C17B	1.4215 (16)
N4A—H4NA	0.90 (2)	N4B—H4NB	0.87 (2)
C1A—C2A	1.3900 (19)	C1B—C6B	1.384 (2)
C1A—C6A	1.3938 (19)	C1B—C2B	1.393 (2)
C1A—H1AA	0.9500	C1B—H1BA	0.9500
C2A—C3A	1.385 (2)	C2B—C3B	1.392 (2)
C2A—H2AA	0.9500	C2B—H2BA	0.9500
C3A—C4A	1.380 (2)	C3B—C4B	1.381 (2)
C3A—H3AA	0.9500	C3B—H3BA	0.9500
C4A—C5A	1.394 (2)	C4B—C5B	1.388 (2)
C4A—H4AA	0.9500	C4B—H4BA	0.9500
C5A—C6A	1.390 (2)	C5B—C6B	1.3990 (18)
C5A—H5AA	0.9500	C5B—H5BA	0.9500
C8A—C9A	1.4932 (17)	C8B—C9B	1.4879 (18)
C9A—C10A	1.3960 (18)	C9B—C14B	1.3946 (17)
C9A—C14A	1.4005 (18)	C9B—C10B	1.3982 (18)
C10A—C11A	1.3907 (19)	C10B—C11B	1.3898 (19)
C10A—H10A	0.9500	C10B—H10B	0.9500
C11A—C12A	1.386 (2)	C11B—C12B	1.3906 (18)
C11A—H11A	0.9500	C11B—H11B	0.9500
C12A—C13A	1.3992 (19)	C12B—C13B	1.3933 (18)
C12A—H12A	0.9500	C12B—H12B	0.9500
C13A—C14A	1.3958 (17)	C13B—C14B	1.3946 (17)
C13A—C15A	1.4977 (18)	C13B—C15B	1.4880 (17)
C14A—H14A	0.9500	C14B—H14B	0.9500
C17A—C22A	1.392 (2)	C17B—C18B	1.3856 (18)
C17A—C18A	1.3958 (19)	C17B—C22B	1.3942 (18)
C18A—C19A	1.388 (2)	C18B—C19B	1.3947 (18)
C18A—H18A	0.9500	C18B—H18B	0.9500
C19A—C20A	1.383 (2)	C19B—C20B	1.386 (2)
C19A—H19A	0.9500	C19B—H19B	0.9500
C20A—C21A	1.393 (2)	C20B—C21B	1.387 (2)
C20A—H20A	0.9500	C20B—H20B	0.9500
C21A—C22A	1.392 (2)	C21B—C22B	1.3916 (18)
C21A—H21A	0.9500	C21B—H21B	0.9500
C22A—H22A	0.9500	C22B—H22B	0.9500
			
C7A—N1A—C6A	131.12 (12)	C7B—N1B—C6B	131.26 (12)
C7A—N1A—H1NA	114.1 (13)	C7B—N1B—H1NB	113.1 (14)
C6A—N1A—H1NA	114.7 (13)	C6B—N1B—H1NB	115.6 (13)
C8A—N2A—C7A	128.61 (11)	C8B—N2B—C7B	128.08 (11)
C8A—N2A—H2NA	116.9 (13)	C8B—N2B—H2NB	115.2 (14)
C7A—N2A—H2NA	114.4 (13)	C7B—N2B—H2NB	116.6 (14)
C15A—N3A—C16A	127.99 (11)	C15B—N3B—C16B	127.96 (11)
C15A—N3A—H3NA	118.0 (13)	C15B—N3B—H3NB	115.5 (14)
C16A—N3A—H3NA	114.0 (13)	C16B—N3B—H3NB	116.5 (14)
C16A—N4A—C17A	128.96 (12)	C16B—N4B—C17B	128.47 (11)
C16A—N4A—H4NA	116.6 (13)	C16B—N4B—H4NB	114.5 (13)
C17A—N4A—H4NA	114.4 (13)	C17B—N4B—H4NB	114.9 (13)
C2A—C1A—C6A	120.28 (14)	C6B—C1B—C2B	119.28 (13)
C2A—C1A—H1AA	119.9	C6B—C1B—H1BA	120.4
C6A—C1A—H1AA	119.9	C2B—C1B—H1BA	120.4
C3A—C2A—C1A	120.24 (14)	C3B—C2B—C1B	120.57 (15)
C3A—C2A—H2AA	119.9	C3B—C2B—H2BA	119.7
C1A—C2A—H2AA	119.9	C1B—C2B—H2BA	119.7
C4A—C3A—C2A	119.32 (13)	C4B—C3B—C2B	119.95 (14)
C4A—C3A—H3AA	120.3	C4B—C3B—H3BA	120.0
C2A—C3A—H3AA	120.3	C2B—C3B—H3BA	120.0
C3A—C4A—C5A	121.22 (15)	C3B—C4B—C5B	119.92 (14)
C3A—C4A—H4AA	119.4	C3B—C4B—H4BA	120.0
C5A—C4A—H4AA	119.4	C5B—C4B—H4BA	120.0
C6A—C5A—C4A	119.37 (14)	C4B—C5B—C6B	120.07 (14)
C6A—C5A—H5AA	120.3	C4B—C5B—H5BA	120.0
C4A—C5A—H5AA	120.3	C6B—C5B—H5BA	120.0
C5A—C6A—C1A	119.56 (12)	C1B—C6B—C5B	120.19 (12)
C5A—C6A—N1A	125.23 (12)	C1B—C6B—N1B	124.44 (12)
C1A—C6A—N1A	115.21 (12)	C5B—C6B—N1B	115.22 (12)
N1A—C7A—N2A	115.00 (11)	N1B—C7B—N2B	114.03 (11)
N1A—C7A—S1A	128.26 (10)	N1B—C7B—S1B	127.76 (10)
N2A—C7A—S1A	116.74 (9)	N2B—C7B—S1B	118.21 (9)
O1A—C8A—N2A	122.67 (12)	O1B—C8B—N2B	123.30 (12)
O1A—C8A—C9A	122.15 (12)	O1B—C8B—C9B	120.59 (12)
N2A—C8A—C9A	115.18 (11)	N2B—C8B—C9B	116.07 (11)
C10A—C9A—C14A	120.18 (12)	C14B—C9B—C10B	120.33 (12)
C10A—C9A—C8A	122.27 (11)	C14B—C9B—C8B	120.58 (11)
C14A—C9A—C8A	117.55 (11)	C10B—C9B—C8B	118.70 (11)
C11A—C10A—C9A	120.28 (12)	C11B—C10B—C9B	120.02 (12)
C11A—C10A—H10A	119.9	C11B—C10B—H10B	120.0
C9A—C10A—H10A	119.9	C9B—C10B—H10B	120.0
C12A—C11A—C10A	119.56 (13)	C10B—C11B—C12B	119.78 (12)
C12A—C11A—H11A	120.2	C10B—C11B—H11B	120.1
C10A—C11A—H11A	120.2	C12B—C11B—H11B	120.1
C11A—C12A—C13A	120.69 (12)	C11B—C12B—C13B	120.11 (12)
C11A—C12A—H12A	119.7	C11B—C12B—H12B	119.9
C13A—C12A—H12A	119.7	C13B—C12B—H12B	119.9
C14A—C13A—C12A	119.85 (12)	C12B—C13B—C14B	120.44 (11)
C14A—C13A—C15A	124.20 (12)	C12B—C13B—C15B	122.76 (11)
C12A—C13A—C15A	115.92 (11)	C14B—C13B—C15B	116.80 (11)
C13A—C14A—C9A	119.34 (12)	C9B—C14B—C13B	119.13 (11)
C13A—C14A—H14A	120.3	C9B—C14B—H14B	120.4
C9A—C14A—H14A	120.3	C13B—C14B—H14B	120.4
O2A—C15A—N3A	122.35 (12)	O2B—C15B—N3B	123.07 (11)
O2A—C15A—C13A	119.77 (12)	O2B—C15B—C13B	121.40 (11)
N3A—C15A—C13A	117.87 (11)	N3B—C15B—C13B	115.51 (11)
N4A—C16A—N3A	114.43 (11)	N4B—C16B—N3B	115.10 (11)
N4A—C16A—S2A	127.03 (10)	N4B—C16B—S2B	127.79 (10)
N3A—C16A—S2A	118.54 (9)	N3B—C16B—S2B	117.11 (9)
C22A—C17A—C18A	120.25 (13)	C18B—C17B—C22B	119.87 (12)
C22A—C17A—N4A	123.49 (12)	C18B—C17B—N4B	124.05 (12)
C18A—C17A—N4A	116.05 (12)	C22B—C17B—N4B	116.07 (12)
C19A—C18A—C17A	119.93 (14)	C17B—C18B—C19B	119.62 (13)
C19A—C18A—H18A	120.0	C17B—C18B—H18B	120.2
C17A—C18A—H18A	120.0	C19B—C18B—H18B	120.2
C20A—C19A—C18A	120.28 (14)	C20B—C19B—C18B	120.66 (13)
C20A—C19A—H19A	119.9	C20B—C19B—H19B	119.7
C18A—C19A—H19A	119.9	C18B—C19B—H19B	119.7
C19A—C20A—C21A	119.58 (13)	C19B—C20B—C21B	119.51 (12)
C19A—C20A—H20A	120.2	C19B—C20B—H20B	120.2
C21A—C20A—H20A	120.2	C21B—C20B—H20B	120.2
C22A—C21A—C20A	120.86 (14)	C20B—C21B—C22B	120.21 (13)
C22A—C21A—H21A	119.6	C20B—C21B—H21B	119.9
C20A—C21A—H21A	119.6	C22B—C21B—H21B	119.9
C21A—C22A—C17A	119.05 (13)	C21B—C22B—C17B	120.00 (13)
C21A—C22A—H22A	120.5	C21B—C22B—H22B	120.0
C17A—C22A—H22A	120.5	C17B—C22B—H22B	120.0
			
C6A—C1A—C2A—C3A	0.0 (2)	C6B—C1B—C2B—C3B	0.1 (2)
C1A—C2A—C3A—C4A	−0.1 (3)	C1B—C2B—C3B—C4B	−0.9 (3)
C2A—C3A—C4A—C5A	0.0 (3)	C2B—C3B—C4B—C5B	0.8 (3)
C3A—C4A—C5A—C6A	0.3 (3)	C3B—C4B—C5B—C6B	0.1 (2)
C4A—C5A—C6A—C1A	−0.4 (2)	C2B—C1B—C6B—C5B	0.9 (2)
C4A—C5A—C6A—N1A	−179.49 (15)	C2B—C1B—C6B—N1B	176.40 (14)
C2A—C1A—C6A—C5A	0.2 (2)	C4B—C5B—C6B—C1B	−1.0 (2)
C2A—C1A—C6A—N1A	179.43 (14)	C4B—C5B—C6B—N1B	−176.88 (14)
C7A—N1A—C6A—C5A	−7.9 (2)	C7B—N1B—C6B—C1B	25.2 (2)
C7A—N1A—C6A—C1A	172.97 (14)	C7B—N1B—C6B—C5B	−159.13 (15)
C6A—N1A—C7A—N2A	179.98 (13)	C6B—N1B—C7B—N2B	−174.40 (13)
C6A—N1A—C7A—S1A	−0.6 (2)	C6B—N1B—C7B—S1B	6.4 (2)
C8A—N2A—C7A—N1A	16.0 (2)	C8B—N2B—C7B—N1B	−4.6 (2)
C8A—N2A—C7A—S1A	−163.55 (11)	C8B—N2B—C7B—S1B	174.65 (11)
C7A—N2A—C8A—O1A	−9.8 (2)	C7B—N2B—C8B—O1B	−8.0 (2)
C7A—N2A—C8A—C9A	170.61 (13)	C7B—N2B—C8B—C9B	169.91 (12)
O1A—C8A—C9A—C10A	158.89 (14)	O1B—C8B—C9B—C14B	132.42 (14)
N2A—C8A—C9A—C10A	−21.56 (19)	N2B—C8B—C9B—C14B	−45.53 (18)
O1A—C8A—C9A—C14A	−20.53 (19)	O1B—C8B—C9B—C10B	−40.45 (19)
N2A—C8A—C9A—C14A	159.02 (12)	N2B—C8B—C9B—C10B	141.60 (13)
C14A—C9A—C10A—C11A	−3.3 (2)	C14B—C9B—C10B—C11B	1.9 (2)
C8A—C9A—C10A—C11A	177.33 (13)	C8B—C9B—C10B—C11B	174.82 (13)
C9A—C10A—C11A—C12A	1.2 (2)	C9B—C10B—C11B—C12B	−3.6 (2)
C10A—C11A—C12A—C13A	1.9 (2)	C10B—C11B—C12B—C13B	1.2 (2)
C11A—C12A—C13A—C14A	−3.0 (2)	C11B—C12B—C13B—C14B	2.8 (2)
C11A—C12A—C13A—C15A	179.14 (14)	C11B—C12B—C13B—C15B	−177.36 (13)
C12A—C13A—C14A—C9A	0.9 (2)	C10B—C9B—C14B—C13B	2.1 (2)
C15A—C13A—C14A—C9A	178.63 (13)	C8B—C9B—C14B—C13B	−170.64 (12)
C10A—C9A—C14A—C13A	2.2 (2)	C12B—C13B—C14B—C9B	−4.50 (19)
C8A—C9A—C14A—C13A	−178.41 (12)	C15B—C13B—C14B—C9B	175.69 (12)
C16A—N3A—C15A—O2A	5.0 (2)	C16B—N3B—C15B—O2B	13.1 (2)
C16A—N3A—C15A—C13A	−175.77 (13)	C16B—N3B—C15B—C13B	−168.35 (12)
C14A—C13A—C15A—O2A	−160.83 (14)	C12B—C13B—C15B—O2B	−153.15 (13)
C12A—C13A—C15A—O2A	17.0 (2)	C14B—C13B—C15B—O2B	26.65 (18)
C14A—C13A—C15A—N3A	20.0 (2)	C12B—C13B—C15B—N3B	28.24 (18)
C12A—C13A—C15A—N3A	−162.26 (13)	C14B—C13B—C15B—N3B	−151.96 (12)
C17A—N4A—C16A—N3A	172.02 (13)	C17B—N4B—C16B—N3B	167.44 (12)
C17A—N4A—C16A—S2A	−7.3 (2)	C17B—N4B—C16B—S2B	−12.7 (2)
C15A—N3A—C16A—N4A	−0.7 (2)	C15B—N3B—C16B—N4B	−13.00 (19)
C15A—N3A—C16A—S2A	178.72 (11)	C15B—N3B—C16B—S2B	167.09 (11)
C16A—N4A—C17A—C22A	−34.1 (2)	C16B—N4B—C17B—C18B	39.0 (2)
C16A—N4A—C17A—C18A	151.23 (14)	C16B—N4B—C17B—C22B	−142.51 (14)
C22A—C17A—C18A—C19A	1.0 (2)	C22B—C17B—C18B—C19B	2.7 (2)
N4A—C17A—C18A—C19A	175.86 (13)	N4B—C17B—C18B—C19B	−178.90 (13)
C17A—C18A—C19A—C20A	0.9 (2)	C17B—C18B—C19B—C20B	0.7 (2)
C18A—C19A—C20A—C21A	−1.8 (2)	C18B—C19B—C20B—C21B	−2.7 (2)
C19A—C20A—C21A—C22A	0.9 (2)	C19B—C20B—C21B—C22B	1.3 (2)
C20A—C21A—C22A—C17A	1.0 (2)	C20B—C21B—C22B—C17B	2.1 (2)
C18A—C17A—C22A—C21A	−1.9 (2)	C18B—C17B—C22B—C21B	−4.1 (2)
N4A—C17A—C22A—C21A	−176.41 (13)	N4B—C17B—C22B—C21B	177.38 (13)

### Hydrogen-bond geometry (Å, º)

**Table:**

*D*—H···*A*	*D*—H	H···*A*	*D*···*A*	*D*—H···*A*
N1*A*—H1*NA*···O1*A*	0.83 (2)	1.976 (19)	2.6722 (16)	140.9 (19)
N2*A*—H2*NA*···S2*B*^i^	0.85 (2)	2.59 (2)	3.4201 (12)	165.0 (19)
N3*A*—H3*NA*···O2*B*^ii^	0.864 (19)	2.31 (2)	2.9715 (15)	133.3 (18)
N4*A*—H4*NA*···O2*A*	0.90 (2)	1.86 (2)	2.6064 (17)	138.2 (18)
N1*B*—H1*NB*···O1*B*	0.86 (2)	1.88 (2)	2.6248 (17)	144.5 (19)
N2*B*—H2*NB*···S1*B*^iii^	0.834 (19)	2.71 (2)	3.4961 (12)	158.3 (19)
N3*B*—H3*NB*···S1*A*^i^	0.84 (2)	2.62 (2)	3.4336 (12)	163.0 (18)
N4*B*—H4*NB*···O2*B*	0.87 (2)	1.92 (2)	2.6543 (16)	141.1 (19)
C5*A*—H5*AA*···S1*A*	0.95	2.51	3.1910 (16)	129
C1*B*—H1*BA*···S1*B*	0.95	2.68	3.2693 (15)	121
C4*B*—H4*BA*···O2*A*^iv^	0.95	2.55	3.4819 (18)	165
C10*B*—H10*B*···S2*A*^v^	0.95	2.84	3.4570 (15)	123
C11*B*—H11*B*···S2*A*^v^	0.95	2.85	3.4687 (15)	123
C14*A*—H14*A*···O2*B*^ii^	0.95	2.35	3.2700 (17)	164
C14*B*—H14*B*···O1*A*^ii^	0.95	2.36	3.2897 (17)	165

Symmetry codes: (i) −*x*+1, −*y*+2, −*z*; (ii) −*x*+1, −*y*+1, −*z*; (iii) −*x*, −*y*+1, −*z*; (iv) −*x*+1, −*y*+1, −*z*+1; (v) *x*−1, *y*+1, *z*.

## References

[bb1] Bruker (2009). *APEX2*, *SAINT* and *SADABS* Bruker AXS Inc., Madison, Wisconsin, USA.

[bb2] Fernandez, E. R., Manzano, J. L., Benito, J. J., Hermosa, R., Monte, E. & Criado, J. J. (2005). *J. Inorg. Biochem.* **99**, 1559–1572. 10.1016/j.jinorgbio.2005.05.004 16005979

[bb3] Kucukguzel, I., Tatar, E., Kucukguzel, S. G., Rollas, S. & Clercq, E. D. (2008). *Eur. J. Med. Chem.* **43**, 381–392. 10.1016/j.ejmech.2007.04.010 17583388

[bb4] Madan, V. K., Taneja, A. D. & Kudesia, V. P. (1991). *J. Indian Chem. Soc.* **68**, 471–472.

[bb5] Rauf, M. K., Din, I. U., Badshah, A., Geilen, M., Ebihara, M., Vos, D. & Ahmed, S. (2009). *J. Inorg. Biochem.* **103**, 1135–1144. 10.1016/j.jinorgbio.2009.05.014 19570580

[bb6] Saeed, A., Abbas, N., Rafique, H., Rashid, S. & Hameed, A. (2009). *Chemistry*, **18**, 152–158.

[bb7] Sheldrick, G. M. (2008). *Acta Cryst.* A**64**, 112–122. 10.1107/S0108767307043930 18156677

[bb8] Spek, A. L. (2009). *Acta Cryst.* D**65**, 148–155. 10.1107/S090744490804362X PMC2631630 19171970

